# Electrospun PVA/Bentonite Nanocomposites Mats for Drug Delivery

**DOI:** 10.3390/ma10121448

**Published:** 2017-12-20

**Authors:** Mariola Ferrández-Rives, Ángela Aurora Beltrán-Osuna, José Antonio Gómez-Tejedor, José Luis Gómez Ribelles

**Affiliations:** 1 Center for Biomaterials and Tissue Engineering, CBIT, Universitat Politècnica de València, 46022 Valencia, Spain; mariolamfr40@gmail.com; 2 Grupo de Procesos Químicos y Bioquímicos, Departamento de Ingeniería Química y Ambiental, Universidad Nacional de Colombia-KR 30 45-03, Bogotá 111321, Colombia; aabeltrano@unal.edu.co; 3 Center for Biomaterials and Tissue Engineering, CBIT, Universitat Politècnica de València, Networking Research Center on Bioengineering, Biomaterials and Nanomedicine (CIBER-BBN), 46022 Valencia, Spain; jogomez@fis.upv.es

**Keywords:** polyvinyl alcohol, electrospinning, crosslinking, nanoclay, drug delivery

## Abstract

Electrospun mats and films of polyvinyl alcohol (PVA) hydrogel are produced for drug delivery. To provide mechanical consistency to the gel a reinforcement by nanoclays is introduced in the polymer matrix. Four different suspensions of nanoparticles in the polymer solution are prepared in an adequate solvent. These suspensions are subjected to an electrospinning process to produce the nanofiber mat, while films are produced by casting. The influence of the process parameters over the nanofibers microstructure is analyzed by scanning electron microscopy (SEM). The effectiveness of nanoclay encapsulation in the nanocomposites is tested by a thermogravimetric analysis. A crosslinking reaction in solution is carried out to prevent the dissolution of the nanocomposites in aqueous media. A model protein (bovine serum albumin, BSA) is absorbed in the nanocomposites to characterize the release kinetics in phosphate-buffered saline (PBS).

## 1. Introduction

Poly(vinyl alcohol) (PVA) is a water-soluble polymer that has attracted particular interest due to its hydrophilicity and biocompatibility properties. PVA is nontoxic and has good thermal stability, making it a promising candidate to be used in biomedicine and biotechnology fields [1]. With this aim, electrospinning has been proven to be a convenient and versatile method to produce non-woven fibrous mats that may be used as nanocomposites in a variety of applications such as filtration membranes, wound-dressing materials, drug delivery systems, and tissue-engineering scaffolds, among others [2]. The electrospinning method gathers lot of interest in tissue engineering as it can produce membrane structures that mimic extracellular matrix (ECM) of native tissue [3]. Electrospun mats can be used as drug delivery reservoirs for controlled and timely local release of drugs, and other molecules to the site of tissue repair [4]. During the electrospinning process, a high voltage is applied to create an electric field between a droplet of polymer solution at the tip of a needle and a collector plate, resulting in an accumulation of charge in the drop and producing a repulsion of the charges within the solution. After a voltage critical value is exceeded, the electrical forces at the surface of a polymer solution overcome the surface tension and a continuous jet of the polymer solution is ejected, and the fibers are collected forming the electrospun mat [5,6]. For this process, PVA can be dissolved in different solvents. One of the most used is deionized water, which does not contain ions [7,8]. However, in such a case, it is possible that the solution does not present the necessary conductivity to be charged when a certain voltage is applied. This issue may be solved by using distilled water which contains more ions than deionized water [9]. Furthermore, the addition of an amount of salt, for example, sodium chloride, and iron nitrate allows the solution to charge properly so the electrospun mats can be formed [10]. In addition, a mixture of several components may be used as a solvent, e.g., a mixture of glacial acetic acid and distilled water, in a volume ratio of 70/30 has been also tested as a solvent of PVA [11].

According to the final application, PVA nanocomposites may be cross-linked physically or chemically to avoid the dissolution of the hydrogel when immersed in water or aqueous solutions. Physical crosslinking can be performed by freeze-thaw cycles in which the starting solution freezes and thaws to introduce crystalline regions that act as crosslinking agents of the chains [12]. However, this type of crosslinking is not permanent and the crystallization is partial, which limits their use in applications that require insolubility, like in drug delivery systems. By contrast, chemical crosslinking is performed by adding an agent that chemically reacts with the polymer chains, so that insoluble systems that act as hydrogels will be created, with capacity of absorbing water. There are different ways to perform a chemical crosslinking. It is possible to add the cross-linker directly into the starting solution prior to performing the electrospinning [7]. Additionally, samples can be immersed in a solution containing crosslinking agents or simply crisscross by applying ultraviolet light [13,14]. It is also possible to perform the crosslinking in vapor phase, e.g., by introducing the electrospun mat into a container with glutaraldehyde, a crosslinking agent which reacts with the hydroxyl groups of the chains of PVA [8,15,16].

On the other hand, hybrid composites based on organic polymers and inorganic materials have also attracted great interest in literature due to the potential enhancement in thermal and mechanic properties of the composite [17,18,19]. A variety of polymer/nanoclay systems has been studied, especially that with layered silicates such us bentonite [20,21]. It has been established that such polymer/nanoclay systems have reinforced mechanical properties, either if the polymer matrix is a hydrogel as in our case or a stiffer material [11,22,23,24,25]. These nanoclay particles are in the form of layers or sheets that can be totally or partially dispersed into the polymer matrix getting a uniform distribution inside the polymer. However, when these nanoclays are incorporated to a polymer matrix, these nanosheets can be interspersed or exfoliated depending basically on the polymer/nanoclay ratio [26]. One of the simplest methods to exfoliate these nanoclays is to dissolve the nanoclay in distilled water [11,26,27]. Furthermore, in many cases ultrasounds are applied to exfoliate nanoclay [28,29].

Studies on the influence of PVA molecular weight as well as the influence of electrospinning parameters on the morphology of PVA electrospun mats are found in literature [30,31,32,33,34]. However, the addition of nanoclays in PVA electrospun composites has been reported mostly only in the last few years. Thus, montmorillonite (MMT) nanoclay has been incorporated in neat PVA and other systems such as polysacharide/PVA/MMT, as super absorbents for personal hygiene products; sericin/PVA/clay (Cloisite 30B) as a medium for antimicrobial air filtration masks or in chitosan/PVA/MMT nanocomposites for biomedical applications [11,35,36,37,38].

In this research, electrospun mats and films of PVA were produced, and then cross-linked by using a glutaraldehyde/sulfuric acid/decahydrate sodium sulfate solution. Hydrophilic bentonite, consisting mostly of montmorillonite (MMT) was added to PVA solution in different contents (0, 2, 10 and 40 wt %) as mechanical reinforcement but also to modulate the kinetic release of a model protein. Bovine serum albumin (BSA) has been used here as a model protein. BSA has been frequently used to test protein delivery from electrospun carriers (see for instance references [39,40,41]). Scanning electron microscopy (SEM) was used to evaluate the influence of the different processing parameters on mean diameter and diameter distributions of PVA electro spun fibers, and to evaluate the morphologies of the PVA/MMT nanocomposites. Thermogravimetric measurements (TGA) were performed to calculate the amount of BSA absorbed by the electrospun mats and the nanoclay contents in films and mats. Finally, swelling tests and absorption and release assays of BSA were performed in phosphate-buffered saline (PBS) to evaluate the behavior of the PVA electrospun mats and films for drug delivery applications. This paper shows the ability to incorporate large amounts of MMT nanoparticles into the submicrometric fibrils of an electrospun mat with high effectiveness and that the nanocomposite mat structure can be fixed by cross-linking the polymeric hydrogel after the electrospinning process. A systematic study of fibrils diameter and homogeneity as a function of processing parameters and MMT contents has been performed.

## 2. Results

### 2.1. Influence of the Processing Parameters on PVA Electrospun Fibers Mean Diameter

**Figure 1:**
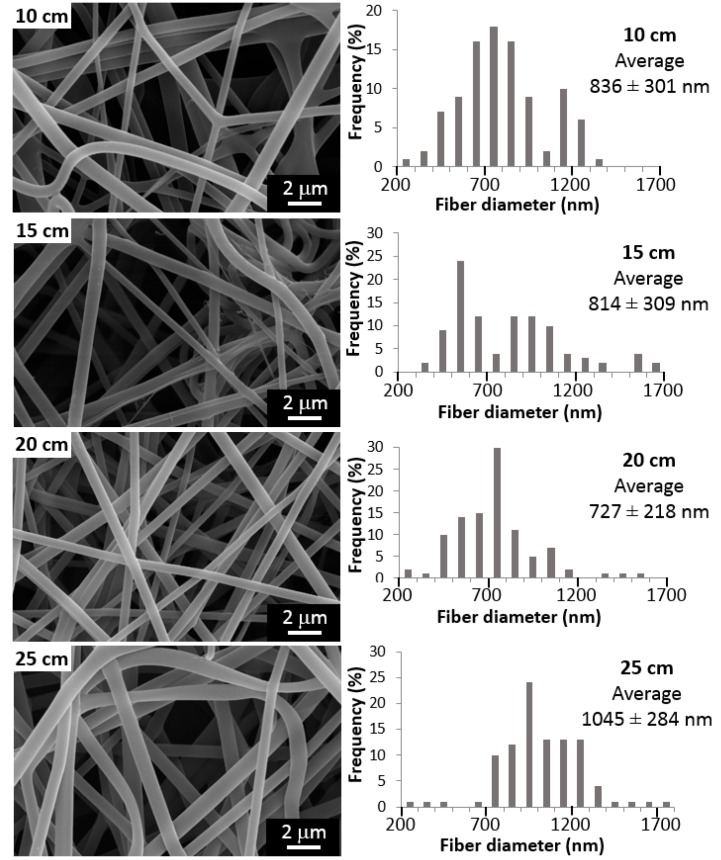
Influence of the tip-to-collector distance on morphology and diameter of electrospun fibers (voltage 20 kV, injection flow rate 4 mL/h).

In order to evaluate the effect of the electrospinning parameters (distance, voltage, and injection flow rate) on the morphology of the samples and the diameter of the fibers, samples were observed and analyzed by SEM. Figure 1 shows the micrographs obtained for PVA electrospun fibers with varying tip-to-collector distance: 10, 15, 20, and 25 cm (at a constant voltage of 20 kV and an injection flow rate of 4 mL/h). It is clear that the relationship between these two parameters is not linear, obtaining a minimum value of 727 nm for a distance of 20 cm. 

**Figure 2:**
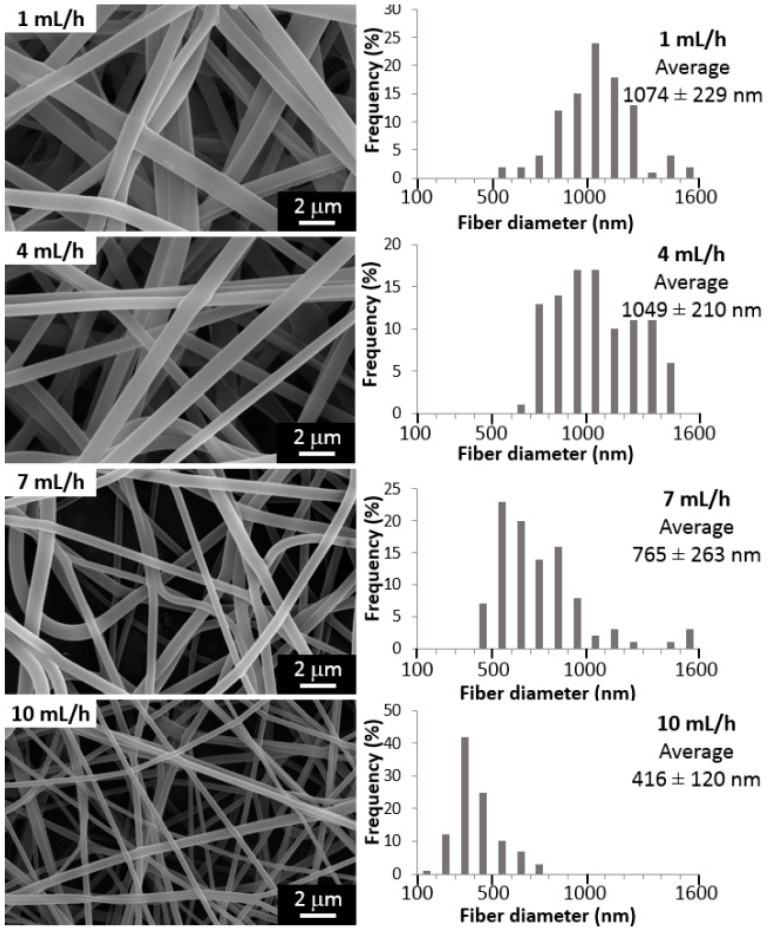
Influence of injection flow rate on morphology and diameter of electrospun fibers (voltage 20 kV, tip-to-collector distance 20 cm).

The feeding rate of the polymer solution may be also examined as another approach to control the morphology and diameters of the fibers. Figure 2 shows the effect of the variation of injection flow rate between 1 and 10 mL/h on the final fiber diameter (voltage 20 kV, tip-to-collector distance 20 cm). It is expected that a minimum injection rate must be kept, to obtain a stable Taylor cone, but also, this feed rate value should be low enough to allow evaporation of the solvent. If not, increasing the injection rate could lead to thicker fibers exhibiting more beads and defects [42]. We have found that the fiber diameter and the flow rate are inversely proportional (Figure 2), so the lowest average diameter (410 nm) is obtained at the highest feed rate (10 mL/h). 

**Figure 3:**
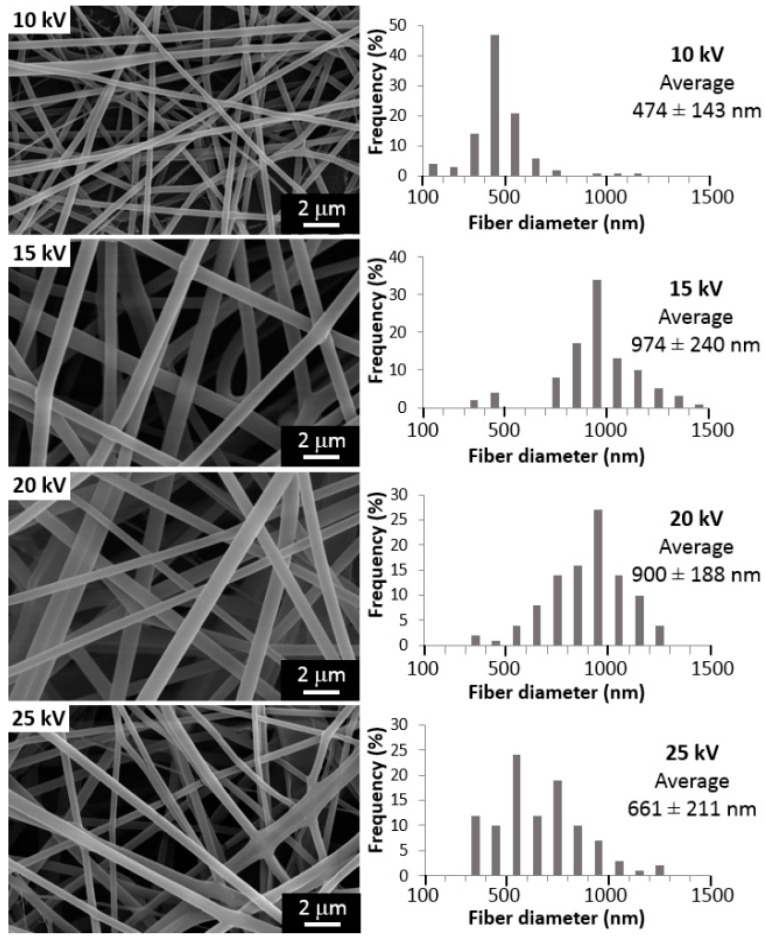
Influence of applied voltage on morphology and diameter of electrospun fibers (tip-to-collector distance 20 cm, injection flow rate of 4 mL/h).

The effect of applied voltage on fiber characteristics is one of the most significant parameters in electrospinning process. Figure 3 shows the morphology and fiber diameter when the voltage is changed from 10 to 25 kV (tip-to-collector distance 20 cm, injection flow rate of 4 mL/h). As it is shown in Figure 3, there is a direct relationship between both parameters, so increasing twice the voltage (from 10 to 20 kV) causes a doubling in the fiber diameter (from 474 to 900 nm). For higher voltages, the average diameter decreases (661 nm). Therefore, more homogenous fibers are formed at a voltage of 20 kV.

For all tests, a *p*-value of less than 0.05 was obtained from Analysis of variance, ANOVA, showing the statistical significance of each factor. Hence, it may be concluded with a 95% confidence level, that the tip-to-collector distance, the voltage and the injection rate have a statistically significant effect on fiber diameter. In summary, the variation of these parameters, as shown in Figure 1, Figure 2 and Figure 3, allowed obtaining always random, continuous and homogeneous fibers, with a smooth surface, and without beads or defects. The effect of the variation of nanoclay contents (%MMT) on fiber characteristics is shown below.

### 2.2. Influence of the Presence of Dispersed Nanoclay

**Figure 4:**
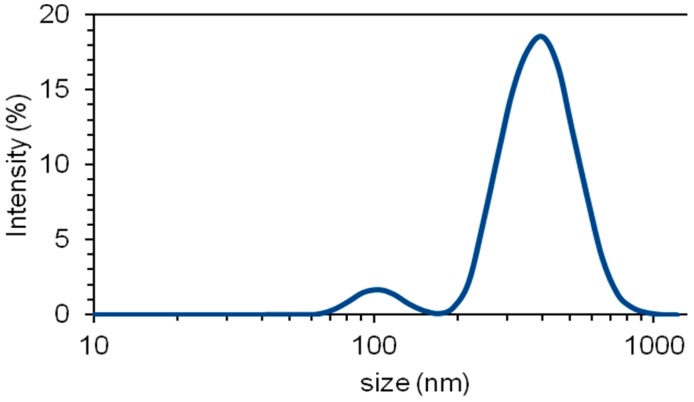
Nanoparticle size distribution for 2% MMT (montmorillonite) sample after sonication treatment.

In another set of experiments, PVA solutions have been reinforced with montmorillonite nanoclay (MMT) in order to potentially enhance the physical properties of electrospun fibers and the mats. Firstly, MMT was dispersed within water under sonication to allow the swelling of the clay, since it has been found that nanoclay proper swelling increases not only its specific area, but also enhances their interactions with the polymeric matrix [43]. Then, no functionalization is needed to mediate between the nanoclay and the polymer, since both, bentonite and PVA are highly hydrophilic, and should interact with each other easily. Thus, a good dispersion and exfoliation of the MMT is expected after the ultrasonication treatment. This was verified for 2% MMT suspensions by DLS measurements as it is shown in Figure 4. It is seen that after the ultrasonication treatment, two peaks were obtained at 408 ± 18 and 104 ± 7 nm for 2% MMT dispersions in water. With 10% MMT dispersions, only one peak was found at 395 ± 46 nm. Multiplicity of peaks were found for 40% MMT dispersions. Thus, it can be expected to have MMT nanoparticles around 400 nm diameter on average with a certain fraction of exfoliated particles in the order of 100 nm. 

**Figure 5:**
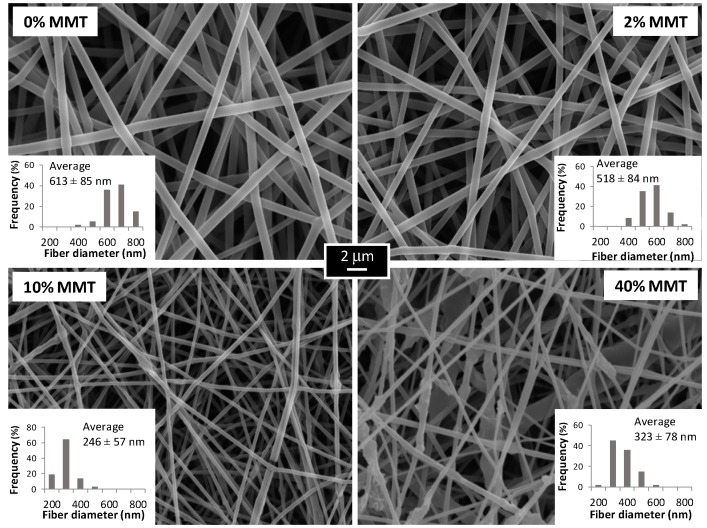
Influence of nanoclay contents (wt %) on morphology and diameter of electrospun fibers (voltage 20 kV, tip-to-collector distance 20 cm, injection flow rate 4 mL/h).

Accordingly, MMT has been incorporated at 2, 10, and 40 wt % in PVA solution to produce electrospun fibers, as shown in Figure 5. It is shown that at 2 wt % MMT uniform, continuous and smooth fibers are formed, while some rippling is observed at higher contents (10 wt %). Since most of the original nanoparticles have a dimension between 400 nm close to the fibril diameter, and thermogravimetric analysis proves that up to 75–85% of the nanoparticles are inside the electrospun fibers, the smooth surface of the fibers proves that nanoparticles are efficiently dispersed along the fibers. Even more, in some cases (10% and 40% MMT) the fibrils in the nanospun mats are thinner than the average diameter of the original particles indicating that additional exfoliation takes place in the jet formation when the MMP suspension is electrospun. A noticeable effect on average diameter is exhibited since an increase of 10% in MMT content causes almost a threefold reduction on fiber diameter (from 613 to 246 nm). In the case of 40 wt % MMT, the average diameter increases again due to the large number of beads and thickenings on the fiber.

Two methods for the measurement of surface tension of PVA/MMT solutions have been applied in this study, and the results are summarized in Table 1. As it is presented in Table 1, the surface tension values obtained for each PVA solution at different MMT contents (0–40 wt %) are quite similar to each other for both methods (29.6–32.4 mN/m and 20.6–26.3 mN/m), the same as with the values of pure solvents (25.2 mN/m for water and 22.3 mN/m for acetic acid) [46].

### 2.3. Thermal Stability and Swelling Behavior of PVA Films and Mats

**Figure 6:**
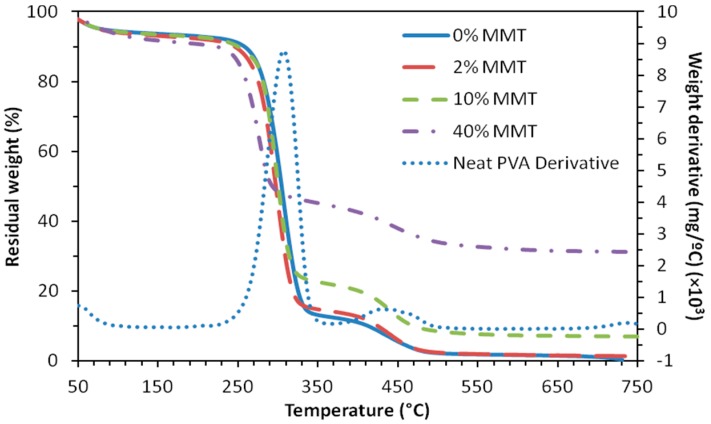
First derivative of TGA (thermogravimetric analysis) for neat PVA (dotted line) and TGA thermograms for PVA mats with different contents of nanoclay: 0% MMT (solid line), 2% MMT (long dashed line), 10% MMT (short dashed line), and 40% MMT (dash dotted line).

The final MMT content on the nanocomposites were determined through thermogravimetric analysis (TGA) as it is shown in Figure 6 for PVA and PVA/MMT electrospun mats. Thermograms for films (not shown) exhibit a similar behavior, and analogous reasoning may be applied. Three peaks are clearly distinguished in Figure 6 for neat PVA films, corresponding to the expected thermal decomposition behavior [27,47,48]. The first broad peak at the beginning is due to the removal of water, which goes from 50 °C to around 150 °C due to the high hydrophilicity of the PVA. Second and third stages correspond to the decomposition of side groups and principal chain, since it has been found that PVA undergoes complete dehydration and depolymerization at around 200 °C and 400 °C, respectively [31]. Residues obtained from each thermogram after 500 °C for both, films and mats, are presented in Table 2. It can be seen that these percentages follow the expected theoretical trend according to the contents of nanoclay. This means that the sample with the higher final residue corresponds to 40 wt % MMT, followed in order by the samples with 10%, 2%, and 0% of nanoclay, respectively. 

It is also shown in Figure 6 that the addition of nanoclay decreases the onset temperature of degradation, therefore the sample with the highest contents of nanoclay (40 wt % MMT) is the one that starts to decompose faster. Likewise, the end of the thermal degradation, around 500 °C, is also shifted to lower values with the increase of MTT contents. Indeed, when the first derivative of each thermogram is plotted against temperature (figure not shown), the principal peak of PVA degradation moves to lower temperatures while increasing the nanoclay contents: 307 °C (0 wt %), 301 °C (2 wt %), 299 °C (10 wt %), and 277 °C (40 wt %).

**Figure 7:**
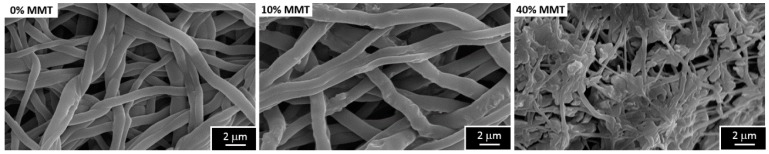
Morphology of crosslinked PVA electrospun nanocomposite mats with different contents of nanoclay.

After the electrospinning process, the electrospun mats were cross-linked to avoid being dissolved in the BSA absorption and delivery assays. Thicker and continuous fibers were obtained for neat PVA mats, and with 2 and 10 wt % of MMT cross-linked samples, with very similar morphologies among them, as shown in Figure 7 as an example for a PVA cross-linked mat with 0 and 10 wt % MTT. This kind of morphology is expected typically when using GA as a crosslinking agent [49]. Only for the sample with the highest contents of nanoclay (40 wt % MMT) big agglomerates are present forming lumps within the fibers, due to the high contents of hydroxyl groups provided by the hydrophilic nanoclay, which may have reacted almost completely with the glutaraldehyde.

**Figure 8:**
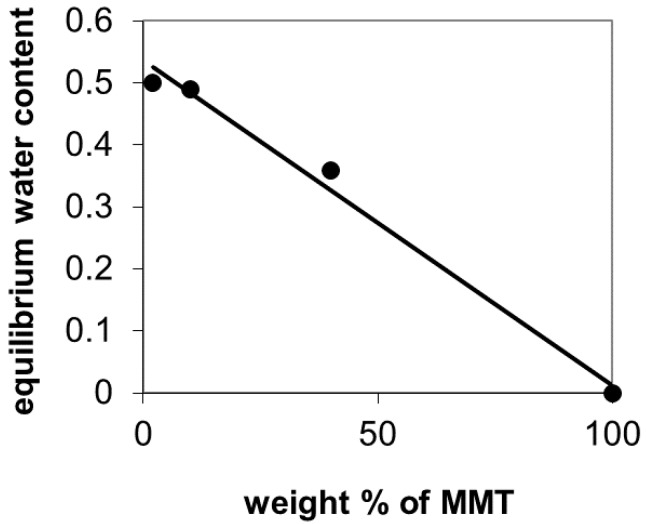
Equilibrium water contents (weight fraction of water in the swollen sample) determined from (by) the swelling ratio and represented against MMT contents.

Moreover, swelling tests for cross-linked PVA mats and films were performed. The weight of all the samples equilibrates after 1 h immersion in water (results not shown). Table 2 shows the equilibrium water contents for films and electrospun mats after 5 h. For films, it is noted that the sample that absorbs more water is the one with contents of nanoclay of 2 wt %, absorbing about 1 gram of water per gram of dry polymer. Next, the sample of 10 wt % of nanoclay absorbs almost 0.9 g of water and finally 40% sample nanoclay, absorbing almost 0.6 g of water per gram of polymer. It was not possible to work with samples of 0 wt % of nanoclay because they were very thin and were broken when handling. The equilibrium water contents (weight fraction of water in the swollen sample) was determined from the swelling ratio and represented against MMT contents in Figure 8. If one assumes that the equilibrium water contents of the MMA nanoparticles is very small compared to that of PVA, the straight line in the figure would correspond to the additivity rule. This means that the filler is not disturbing the swelling on the hydrogel matrix, thus, it is not contributing to increasing the cross-linking density of the gel, which would correspond to experimental points below the straight line in Figure 8. 

In the case of the electrospun mats, the water uptake is higher, around 2.5 grams of water per gram of polymer. Additionally, the water content becomes less dependent on the amount of nanoclay contained by the material.

### 2.4. BSA Absorption and Delivery Assays

**Figure 9:**
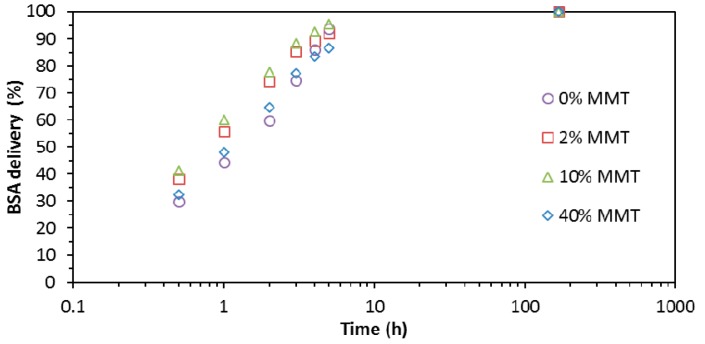
BSA (bovine serum albumin) delivery from PVA electrospun mats for different contents of nanoclay: 0% (circle), 2% (square), 10% (triangle), and 40% MMT (diamond).

The absorption capacity of BSA of the electrospun mats was determined by TGA analysis. The thermogram of the nanocomposites after absorption of BSA and drying, can be expressed as:

(1) $$\frac{dW}{dT}\left(T\right)=X_{\mathrm{BSA}}\frac{dW_{\mathrm{BSA}}}{dT}\left(T\right)+\left(1-X_{\mathrm{BSA}}\right)\frac{dW_{\mathrm{PVA}-\mathrm{MMT}}}{dT}\left(T\right)$$

where *dW*_BSA_/*dT* and *dW*_PVA−MMT_/*dT* are the mass fraction derivative for the thermograms of pure BSA and the electrospun hydrogel with the same MMT contents before BSA absorption, and *X*_BSA_ is the mass fraction of BSA in the sample. The fitting of the experimental TGA data to Equation (1) allowed determining that the mass fraction of BSA in the PVA electrospun mat (after absorption to equilibrium) was 40%. Likewise, for the samples with 2%, 10% and 40% MMT, the mass fractions of BSA found were 40%, 25%, and 20%, respectively. The accumulated time delivery is shown in Figure 9 where the results are normalized to the delivery time of seven days. It can be seen that the differences in the kinetics between the different samples are very small, below the uncertainty of the measurements. 

## 3. Discussion

### 3.1. Influence of the Processing Parameters on PVA Electrospun Fibers Mean Diameter

The diameter of the electrospun fibers had a minimum value for a distance of 20 cm (see Figure 1). This result may be explained by two effects that occur simultaneously causing opposing tendencies. On the one hand, the intensity of the electric field (at constant voltage) will decrease if the tip-to-collector distance is increased. This causes a lower acceleration, meaning less stretched polymer chains and, therefore, the formation of thicker fibers. On the other hand, fibers will have more time to dry, reaching the collector with less solvent and thinner diameters. Thus, the final result depends on the specific solvent-polymer system and its parameters, such as molecular weight, molecular interactions, polymer concentration, and other physicochemical characteristics. Although some authors found no significant effect of the tip-target distance over the fiber morphology [34], the results in this study are in agreement with those obtained for PVA in the majority of reports. That is, there is a minimum diameter value obtained for an optimum distance for each solution that allows the solvent to evaporate before reaching the collector [31,35,38,50]. When working with very small distances, drops at the tip of the needle are formed, leading to a solution drip. Moreover, beads are formed with very small and very large distances [46].

We have found that the fiber diameter decreases when the flow rate increases (Figure 2). This unanticipated behavior could be the result of the chosen combination of values for the other variables (applied voltage and tip-to collector distance) that influence the morphology of the fiber, along with other variables here untested (e.g., polymers solution concentration and molecular weight). Specifically, for PVA, only one study was found where the flow rate variation is evaluated but for very low values (0.1 to 0.3 mL/h) [34]. No clear trend was observed nor the fiber diameters were calculated, and the authors only reported a slight change in morphology, since beads were formed at the highest flow rate (0.3 mL/h). Indeed, many reviews point out the lack of studies investigating the relationship between feed rate and fiber size in a systematical manner [2,46,51,52]. 

One of the parameters that mostly influences the fiber morphology in the electrospinning process is the applied voltage. It is known that a certain threshold voltage must be overcome so that the fibers can be ejected from the Taylor cone to the collector [51]. All the same, for higher voltage values, two phenomena may be exhibited. From one point of view, an increase in the applied voltage causes an increment in the electrostatic repulsive forces in the jet, causing stretching of the polymeric solution, ultimately favoring narrow diameters. On the contrary, due to the higher electric field force, the solution is removed from the tip more quickly as soon as it is ejected from Taylor cone, allowing less time for elongational flow and solvent evaporation, thus increasing the fiber diameter [53]. The overall effect of applied voltage variation will depend on the dominance of one or the other phenomenon, hence causing apparent disagreement on the literature. Some authors reported a clear diminishing of diameter values when the voltage is increased for Nylon and atactic PVA electrospun fibers [31,50]. A few others reported a weak effect of electric field and voltage on fiber diameter for polyethylene oxide (PEO), and poly(hydroxybutyrate-co-valerate) (PHBV) [54,55]. Additionally, for PVA electrospun fibers, a significant increase in the applied voltage (from 7 to 19 kV) causes only a slight decrease (from 310 to 270 nm) in the average diameter [32]. As it is shown in Figure 3, there is a direct relationship between both parameters up to a voltage of 20 kV. For higher voltages, there might be some instabilities in the jet, enhanced by the high molecular weight of the PVA used (130,000 g/mol), that leads to the formation of several jets [51]. These mini-jets originate the ramification morphology of the fibers, as it is observed in Figure 3 (for 25 kV), and this may cause the decrease in the average diameter. Therefore, more homogenous fibers are formed at a voltage of 20 kV, corresponding to the same value found in another study for high molecular a-PVA (176,000 g/mol, at a tip-to-collector distance of 10 cm) [31]. 

This increasing linear trend within the applied voltage and fiber diameter has been corroborated by many studies: between 20 and 30 kV for poly(l-lactic acid) (PLLA, Mw: 100,000 g/mol) and for a wide range of voltages (5–75 kV) for PVA of different molecular weights (14,000–75,000 g/mol) at varied concentrations (7–10 wt % in water) in the last decade [33,34,35,38,56]. These studies support then the premise that when higher voltages are applied, there is more polymer ejected from the tip, facilitating the formation of thicker fibers. 

### 3.2. Influence of the Presence of Dispersed Nanoclay

With a MMT content of 10 wt %, a significant decrease in the diameter of the fibers has been observed (Figure 5). Nowadays, it is well established that, as a general rule, the addition of a filler to a polymeric solution increases its viscosity. Thus, a higher viscosity helps the jet become more resistant to stretching due to the repulsion electrostatic forces, contributing to larger fiber diameters. Certainly, some authors have reported an increase of PVA electrospun fiber diameter with the addition of different kinds of nanoclay: unmodified MMT and Cloisite 30B [35,38]. Nevertheless, quite an opposite behavior has been also found in literature when other nanocomposites such as layered double hydroxides (Mg-Al LDH) have been used for PVA systems, improving the spinnability of the solution, with a clear reduction on the final diameters [57]. In doing so, the authors reported significantly lower fiber diameters (100–200 nm) when compared to neat PVA (400–500 nm) for contents of LDH of 3–7 wt %. Likewise, the addition of 3% MMT has also lead to a reduction of 30% in the fiber diameter on chitosan/PVA systems [11]. In this case, the authors attribute such behavior to a possible lower viscosity of the electrospun solution, due to the preparation protocol followed for the samples For 40 wt % MMT, the average diameter increases again due to the large number of beads and thickenings on the fiber. These beads are due to small aggregates of nanoclay that remain inside the fibers and that decrease the homogeneity of the sample [26,35,48]. These bead-on-string structures might be also caused by the viscosity increment of the polymer solution that causes instabilities in the flow, as a form of resistance of the jet to the extensional flow, thus creating defects on the fibers [58].

Along these lines, the effect of nanoclay contents on average diameters must be considered in detail. Since the addition of layered silicates during electrospinning can change the shear viscosity, the conductivity, and the surface tension of a polymer solution, it is worth the time to analyze some of these three properties [43]. On the one hand, the correlation between zero shear viscosity and electrospun fiber diameters has been already properly studied concluding that a power law dependence of diameter on viscosity is much weaker than expected, and that there are more primary variables such as solution’s electrical properties and surface tension [59]. It also may be noted that a study on high molecular weights (50,000–89,000 g/mol) PVA electrospun nanofibers has shown that for concentrations higher than 9 wt % of PVA, the high viscosity of the solution inhibits solvent evaporation, making the fibers still wet when reaching the collector, and causing a planar morphology of the fibers due to the impact on the collector [30]. However, this kind of morphology is absent for the conditions used in the current study (130,000 g/mol, 8 wt % PVA, Figure 5), indicating that the viscosity effect may not be so significant in this case, and that the jet undergoes appropriate stretching by extensional flow. Besides, it has been found that fiber diameter is almost independent from the viscosity of the spinning solution when enough chain entanglement density is reached (e.g., high molecular weights) for poly(ethylene oxide) PEO solutions with nanoclay Laponite [59].

On the other hand, the influence of surface tension and conductivity on the average diameters is much less studied. In this study we have analyzed the surface tension of the electrospinning solutions (Table 1). The values obtained for the surface tension imply that it is not significantly influenced by the nanoclay contents. Hence, a reasonable explanation of the diameter fiber thinning exhibited upon MMT contents in Figure 5 is that the addition of the nanoclay (which is charged) causes an increment on the charge density of the solution and that these changes on the conductivity of the polymer solution could ease the stretching of the fiber under the applied voltage, favoring thinner diameters [59].

### 3.3. Thermal Stability and Swelling Behavior of PVA Films and Mats

The addition of nanoclay decreases the onset temperature of degradation and the thermal degradation (Figure 6). At this respect, it has been reported that nanoclay addition in polyethylene may have two opposite effects on the thermal stability of the polymer matrix, by improving its barrier characteristics, or by augmenting its catalytic degradation [60]. Then, the catalytic effect of MMT at the beginning of the degradation process could be causing this decrease in thermal stability, which has also been reported for nanoclay delamination by ion exchange of interlayer ions with Heggin ions in PVA/MMT systems and for PVA/LDH nanocomposites when exfoliation was done by using a non-ionic surfactant, Triton X, helped by ultrasonication [36,57].

Final contents of MMT (Table 2) determined by TGA are slightly lower (75% to 85%) than the theoretical contents. Since no MMT particles are shown on the surface of the fibrils in the SEM pictures, these results show that most of the MMT particles have been integrated into the matrix. The difference between the final MMT contents of the mat and the theoretical one can be due to a fraction of MMT particles that were just dispersed between the fibrils and are extracted when immersed in the crosslinking bath, but a certain inhomogeneity in the suspension, which is electrospun, cannot be ruled out since the MMT particles might tend to settle in the syringe during the electrospun process.

Water uptake is higher for the electrospun mats than for the films. This behavior can be explained because, in addition to the water absorbed by the fibrils, liquid water is also filling the pores between fibers. Furthermore, the water contents becomes less dependent on the amount of nanoclay contained by the material, since the water absorbed in the pores is much higher than that absorbed by the material itself. In the sample containing 40 wt % MMT, the water uptake decreases because its porosity is smaller than the rest of samples, as shown in Figure 7. These swelling ratios are consistent with those reported for PVA cross-linked with concentrations from 10–25 wt % of GA to PVA [32].

### 3.4. BSA Absorption and Delivery Assays

The differences in the release kinetics between the different samples are very small, below the uncertainty of the measurements. The values of the total amount of protein delivered are nevertheless, non-reproducible. It seems that an undetermined fraction of the protein remains adsorbed on the surface of the fibrils within the pores of the electrospun mat and it is responsible for the observed delivery at short times.

In conclusion, the content of nanoclay does not influence the release kinetics after seven days. Further measurements are needed for greater release times to determine more accurately the influence of nanoclay contents in the release kinetics of BSA.

## 4. Materials and Methods

### 4.1. Materials

Poly(vinyl alcohol) (PVA, Mw: 130,000 g/mol, 99+% hydrolyzed, Sigma-Aldrich, Saint Louis, MN, USA) was dissolved in a mixture of distilled water and glacial acetic acid (Mw: 60.05 g/mol, Scharlau Chemie, Barcelona, Spain). Hydrophilic bentonite (MMT, montmorillonite nanoclay, Mw: 180.1 g/mol, Sigma-Aldrich) was used as nanofiller. An aqueous crosslinking solution containing glutaraldehyde (GA, 25% aqueous solution, Mw: 100.12 g/mol, Scharlau Chemie), sulfuric acid (95–97% for synthesis, Mw: 98.08 g/mol, Scharlau Chemie) and decahydrate sodium sulfate (Mw: 322.20 g/mol, Sigma-Aldrich) was prepared for the crosslinking reaction of films and mats. Bovine serum albumin (BSA, cold ethanol fraction, pH 5.2, ≥96%, Mw: 66,430 Da, Sigma-Aldrich) was used for drug delivery assays, using phosphate buffer saline (PBS) as solvent, and MilliQ-deionized water (18.2 MΩ cm) was used whenever needed.

### 4.2. Preparation of PVA and PVA/MMT Solutions

An 8 wt % solution was prepared by mixing PVA with a solvent composed by a mixture of distilled water and glacial acetic acid in a volume ratio of 30/70, respectively. The polymer was dissolved by magnetic stirring at 80 °C until complete dissolution. Films and mats containing 0% of MMT were prepared with this solution. In order to incorporate MMT into the solution at 2, 10 and 40 wt %, two aqueous solutions of MMT were prepared at 5 wt % to produce mats and films of 2 and 10 wt % of MMT, and another solution at 23 wt % to produce mats and films of 40 wt % of MMT. The solutions were mixed under sonication (30 min, 100 W) to exfoliate the nanoclay and to homogenize the sample [28]. During this step, the containers were placed in an ice bath to prevent high temperatures generated by the sonicator from interfering with the results. The final temperature reached by the water was 5 °C. After that, they were mixed with a proper quantity of the polymer solution at 8 wt % to produce PVA/MMT solutions that were used to produce films and electrospun mats.

### 4.3. Electrospinning Conditions

Initially, a solution of PVA in deionized and distilled water at concentration of 10% and 6% *w*/*w* were tested, following the protocols of references [8,9,47]. We also tried adding 1% *w*/*v* of sodium chloride to the deionized water with the purpose of increasing ionic conductivity [10]. From these previous experiments, it was found that homogenous fibers without defects were obtained only for PVA solved at 8 wt % in a mixture of distilled water and glacial acetic acid in a volume ratio of 30/70. With this solution, electrospinning mats at different distances, voltages, and injection rates were produced. To determine their effect into the diameter and morphology of the fibers, four different values of distance (10, 15, 20, and 25 cm), voltage (10, 15, 20, and 25 kV) and flow rates (1, 4, 7, and 10 mL/h) were tested. In all cases, a needle of 0.6 mm inner diameter was used. The electrospinning process was performed at room temperature and humidity. The nanofibers were collected on aluminum foils, dried and stored at room temperature until they were used. 

### 4.4. Films Preparation: PVA and PVA/MMT

Solutions of PVA with different contents of MMT were used to prepare films of PVA and PVA/MMT. A small amount of each solution was placed separately in a container under mechanical stirring to evaporate part of the solvent, thickening the solution. This process was carried out in a bath at constant temperature (70–80 °C) and a stirring rate of 350 rpm. Afterwards, the containers were placed in a vacuum desiccator to remove the air and to prevent the formation of bubbles in the film. A small amount of each mixture was softly poured into a Petri dish and spread in a thin layer, around 0.5 mm thick. Finally, the plates were dried at room temperature to evaporate the whole solvent from the film.

### 4.5. Crosslinking of Films and Mats

Films and electrospun mats of PVA and the nanocomposites with different contents of MMT were cut with dimensions of 0.5 × 3 cm. Six replicas of each sample were made: three for controls and three for testing. They were weighed to obtain their mass at the beginning of the assay (*t* = 0 h). The crosslinking reaction was carried out according to a previous protocol by immersing the samples in 400 mL of an aqueous solution composed by glutaraldehyde (0.03 M) as a crosslinking agent, sulfuric acid (0.15 M) as a pH regulator and decahydrate sodium sulfate (0.96 M) to prevent PVA samples from being dissolved in the solution during the crosslinking reaction [13]. This solution was preheated to the crosslinking temperature, 40 °C. Then, the containers with the sample immersed in the crosslinking solution were maintained at 40 °C for 1 h in a thermostatic bath. 

### 4.6. Swelling Assay

Swelling tests for PVA films and mats were performed to evaluate the capacity of water absorption of the hydrogels and to validate the success of the crosslinking reaction. In these tests, each sample was immersed in a Petri dish with deionized water. The samples were removed from the water every hour, dried on absorbent paper to remove surface water, and reintroduced into water after weighing. The test was done until a constant weight for the swollen sample was reached. After completing the assay, samples were dried in vacuum at room temperature and then weighed. The water absorption capacity of each sample was calculated by using the following Equation:

*SR* = (*w_s_* − *w_d_*)/*w_d_* (2)

where *SR* is the swelling ratio (grams of water uptake per gram of dry PVA), and *w_d_* and *w_s_* are the weights of dry and swollen PVA respectively.

### 4.7. Absorption, Release, and Quantification of Protein

To perform the protein absorption assay, 20% of BSA in PBS solution was prepared. Control replicas did not absorb protein while test replicas were immersed separately in 2 mL of the solution for 24 h. During that period, the samples were weighed at 15, 30, 45 min, 1, 2, 3, 4, 5, and 24 h. Thereafter, they were dried in air and then in vacuum. The release of protein was performed at 37 °C with control and test replicas, to take into account a possible absorbance value given by the own material in the wavelength used for testing. For this, the samples were immersed separately in 1 mL of PBS for 5 h, extracting 500 µL of the supernatant at 30 min, 1, 2, 3, 4, 5 h, and seven days. After each extraction, another 500 µL of PBS was added to keep the total volume of 1 mL. For protein quantification, a Micro BCA Protein Assay kit (ThermoFisher Scientific, Waltham, MA, USA) was used. This is a colorimetric method for the detection and quantification of proteins in the range of 20–2000 mg/mL. The absorbance is measured using a VICTOR3 1420 Multilabel Counter equipment (PerkinElmer, Waltham, MA, USA) at a wavelength of 570 nm. 

### 4.8. Characterization

The surface tension of PVA solutions with different concentrations of MMT was determined through two methods: the first method is based on Tate’s law with Lee-Chan-Pogaku correction, while the second one is based on a drop weight analysis of the solution through needles of different gages [44,45,61]. The morphology of the electrospun PVA nanofibers was observed by scanning electron microscopy (SEM) using a Jeol JSM–6300 microscope (Peabody, St. Louis, MS, USA), after a gold-palladium coating. The average diameter and standard deviation of the fibers were determined by analyzing the SEM micrographs with ImageJ software (LOCI, University of Wisconsin-Madison, Madison, WI, USA). The evaluation of the significance of the data obtained after evaluating the effect of the process parameters on the fiber diameter was performed using the ANOVA test with Statgraphics XVI software (Statgraphics Technologies, The Plains, VA, USA). Thermogravimetric analysis (TGA) was carried out for mats and films using a TGA/DSC 2 STAR System (Mettler Toledo, Columbus, OH, USA), with a heat rate of 10 K/min from 30 to 1000 °C under nitrogen gas atmosphere. The nanoclay size distributions were determined by dynamic light scattering (DLS) of the MMT suspensions in water, using a Zetasizer Nano ZS equipment by Malvern Instruments Ltd. (Malvern, UK), with a 4 mW 632.8 nm red laser, at an angle of 175°. Five measurements were made for each sample, using 12 runs of 10 s for each scan, and the average size value is reported for the intensity-based particle size distributions given by Zetasizer software (Malvern Instruments Ltd., Malvern, UK).

## 5. Conclusions

Nanoclay can be successfully distributed in PVA electrospun fibrils up to 40% by weight of nano-filler. It has been found that the diameter of the fibrils decreases in the composite electrospun mats with respect to pure PVA what could be explained by an increase of the conductivity of the PVA solution when bentonite nanoparticles are dispersed. The effectiveness of the inclusion of nanoclay in the electrospun mats, evaluated by TGA was between 75% and 85% depending on the nanoclay content. Water sorption capacity rapidly decreases in the composites in the form of films used as controls when increasing the filler content, nevertheless, in the electrospun mats most of the water that the sample contains is filling the pores between fibrils and the total amount of absorbed water depends only slightly on the sample composition and is mainly due to changes in fibrils morphology at high nanoclay contents. Absorption of albumin was performed by immersion in a protein solution and subsequent drying. The decrease in protein contents with increasing nanoclay fraction in the samples is poorly reproducible and it is ascribed to morphology changes. Kinetics of albumin delivery seems to be independent of nanoclay contents. When the hydrogel is immersed in the protein solution, the amount of liquid filling the pores is higher than that absorbed by the fibrils, thus, after drying, an important part of the protein is adsorbed on the fibrils surfaces and its delivery kinetics becomes independent of the MMT content.

## Figures and Tables

**Table 1:** Density and surface tension values for PVA/MMT (Poly(vinyl alcohol)/montmorillonite) electrospun solutions. Method 1 is based on Tate’s law with Lee-Chan-Pogaku correction and method 2 on drop weight analysis through needles of different gages [44,45].

%	Density	Surface Tension (mN/m)	
MMT	(kg/m^3^)	Method 1	Method 2
0	1076	31.7	20.7
2	1082	32.4	26.3
10	1089	29.6	21.8
40	1097	31.1	20.6

**Table 2:** Residual percentages obtained through TGA and water uptake per gram at 5 h for PVA and PVA/MMT films and mats.

Product	Theoretical Content of MMT (%)	TGA Measured Nanoclay Content (%)	Swelling Ratio at 5 h (g/g)
Films	0	0	---
	2	1.9	1.00
	10	8.4	0.96
	40	35.1	0.56
Electrospun mats	0	0	2.46
	2	1.6	2.52
	10	7.5	2.66
	40	34.5	2.18

## References

[1] Paradossi G., Cavalieri F., Chiessi E., Spagnoli C., Cowman M.K. (2003). Poly(vinyl alcohol) as versatile biomaterial for potential biomedical applications. J. Mater. Sci. Mater. Med..

[2] Sill T.J., von Recum H.A. (2008). Electrospinning: Applications in drug delivery and tissue engineering. Biomaterials.

[3] Suntornnond R., An J., Yeong W.Y., Chua C.K. (2015). Biodegradable Polymeric Films and Membranes Processing and Forming for Tissue Engineering. Macromol. Mater. Eng..

[4] Nguyen L.T.H., Chen S., Elumalai N.K., Prabhakaran M.P., Zong Y., Vijila C., Allakhverdiev S.I., Ramakrishna S. (2013). Biological, Chemical, and Electronic Applications of Nanofibers. Macromol. Mater. Eng..

[5] Gómez-Tejedor J.A., Van Overberghe N., Rico P., Ribelles J.L.G. (2011). Assessment of the parameters influencing the fiber characteristics of electrospun poly(ethyl methacrylate) membranes. Eur. Polym. J..

[6] Silva C.S.R., Luz G.M., Gamboa-martÍnez T.C., Mano J.F., GÓmez ribelles J.L., GÓmez-tejedor J.A. (2014). Poly(ɛ-caprolactone) Electrospun Scaffolds Filled with Nanoparticles. Production and Optimization According to Taguchi’s Methodology. J. Macromol. Sci. Part B.

[7] Nugroho R.W.N., Roy P.K., Odelius K., Albertsson A.-C. (2013). Crosslinked PVAL nanofibers with enhanced long-term stability prepared by single-step electrospinning. Polym. Adv. Technol..

[8] Destaye A.G., Lin C.-K., Lee C.-K. (2013). Glutaraldehyde Vapor Cross-linked Nanofibrous PVA Mat with in Situ Formed Silver Nanoparticles. ACS Appl. Mater. Interfaces.

[9] Pathan S.G., Fitzgerald L.M., Ali S.M., Damrauer S.M., Bide M.J., Nelson D.W., Ferran C., Phaneuf T.M., Phaneuf M.D. (2015). Cytotoxicity associated with electrospun polyvinyl alcohol. J. Biomed. Mater. Res. Part B Appl. Biomater..

[10] Arayanarakul K., Choktaweesap N., Aht-ong D., Meechaisue C., Supaphol P. (2006). Effects of Poly(ethylene glycol), Inorganic Salt, Sodium Dodecyl Sulfate, and Solvent System on Electrospinning of Poly(ethylene oxide). Macromol. Mater. Eng..

[11] Koosha M., Mirzadeh H., Shokrgozar M.A., Farokhi M. (2015). Nanoclay-reinforced electrospun chitosan/PVA nanocomposite nanofibers for biomedical applications. RSC Adv..

[12] Hassan C.M., Peppas N.A. (2000). Structure and Applications of Poly(vinyl alcohol) Hydrogels Produced by Conventional Crosslinking or by Freezing/Thawing Methods. Biopolymers PVA Hydrogels, Anionic Polymerisation Nanocomposites.

[13] Kim K.-J., Lee S.-B., Han N.W. (1993). Effects of the Degree of Crosslinking on Properties of Poly(vinyl alcohol) Membranes. Polym. J..

[14] Zhang X., Tang K., Zheng X. (2016). Electrospinning and Crosslinking of COL/PVA Nanofiber-microsphere Containing Salicylic Acid for Drug Delivery. J. Bionic Eng..

[15] Lee I.W., Li J., Chen X., Park H.J. (2016). Electrospun poly(vinyl alcohol) composite nanofibers with halloysite nanotubes for the sustained release of sodium d-pantothenate. J. Appl. Polym. Sci..

[16] Tsai R.-Y., Hung S.-C., Lai J.-Y., Wang D.-M., Hsieh H.-J. (2014). Electrospun chitosan-gelatin-polyvinyl alcohol hybrid nanofibrous mats: Production and characterization. J. Taiwan Inst. Chem. Eng..

[17] Rezwan K., Chen Q.Z., Blaker J.J., Boccaccini A.R. (2006). Biodegradable and bioactive porous polymer/inorganic composite scaffolds for bone tissue engineering. Biomaterials.

[18] Vallet-Regí M., Colilla M., González B. (2011). Medical applications of organic-inorganic hybrid materials within the field of silica-based bioceramics. Chem. Soc. Rev..

[19] Armentano I., Dottori M., Fortunati E., Mattioli S., Kenny J.M. (2010). Biodegradable polymer matrix nanocomposites for tissue engineering: A review. Polym. Degrad. Stab..

[20] Lee W.-F., Chen Y.-C. (2004). Effect of bentonite on the physical properties and drug-release behavior of poly(AA-co-PEGMEA)/bentonite nanocomposite hydrogels for mucoadhesive. J. Appl. Polym. Sci..

[21] Bhattacharya S.S., Sen K.K., Sen S.O., Banerjee S., Kaity S., Ghosh A.K., Ghosh A. (2011). Synthesis and Characterization of Poly(acrylic acid)/modified Bentonite Superabsorbent Polymer. Int. J. Polym. Mater..

[22] Zerda A.S., Lesser A.J. (2001). Intercalated clay nanocomposites: Morphology, mechanics, and fracture behavior. J. Polym. Sci. Part B Polym. Phys..

[23] Rao Y., Pochan J.M. (2007). Mechanics of Polymer-Clay Nanocomposites. Macromolecules.

[24] Lee J.H., Park T.G., Park H.S., Lee D.S., Lee Y.K., Yoon S.C., Nam J.-D. (2003). Thermal and mechanical characteristics of poly(l-lactic acid) nanocomposite scaffold. Biomaterials.

[25] Marras S.I., Kladi K.P., Tsivintzelis I., Zuburtikudis I., Panayiotou C. (2008). Biodegradable polymer nanocomposites: The role of nanoclays on the thermomechanical characteristics and the electrospun fibrous structure. Acta Biomater..

[26] Islam M.S., Yeum J.H., Das A.K. (2012). Effect of pullulan/poly(vinyl alcohol) blend system on the montmorillonite structure with property characterization of electrospun pullulan/poly(vinyl alcohol)/montmorillonite nanofibers. J. Colloid Interface Sci..

[27] Ji H.M., Lee H.W., Karim M.R., Cheong I.W., Bae E.A., Kim T.H., Islam M.S., Ji B.C., Yeum J.H. (2009). Electrospinning and characterization of medium-molecular-weight poly(vinyl alcohol)/high-molecular-weight poly(vinyl alcohol)/montmorillonite nanofibers. Colloid Polym. Sci..

[28] Kahraman H.T., Gevgilili H., Kalyon D.M., Pehlivan E. (2013). Nanoclay dispersion into a thermosetting binder using sonication and intensive mixing methods. J. Appl. Polym. Sci..

[29] Kaboorani A., Riedl B., Blanchet P. (2013). Ultrasonication Technique: A Method for Dispersing Nanoclay in Wood Adhesives. J. Nanomater..

[30] Koski A., Yim K., Shivkumar S. (2004). Effect of molecular weight on fibrous PVA produced by electrospinning. Mater. Lett..

[31] Lee J.S., Choi K.H., Ghim H.D., Kim S.S., Chun D.H., Kim H.Y., Lyoo W.S. (2004). Role of molecular weight of atactic poly(vinyl alcohol) (PVA) in the structure and properties of PVA nanofabric prepared by electrospinning. J. Appl. Polym. Sci..

[32] Ding B., Kim H.-Y., Lee S.-C., Shao C.-L., Lee D.-R., Park S.-J., Kwag G.-B., Choi K.-J. (2002). Preparation and characterization of a nanoscale poly(vinyl alcohol) fiber aggregate produced by an electrospinning method. J. Polym. Sci. Part B Polym. Phys..

[33] Adomavičiūtė E., Milašius R., Levinskas R. (2007). The Influence of Main Technological Parameters on the Diameter of Poly(vinyl alcohol) (PVA) Nanofibre and Morphology of Manufactured Mat. Mater. Sci..

[34] Zhang C., Yuan X., Wu L., Han Y., Sheng J. (2005). Study on morphology of electrospun poly(vinyl alcohol) mats. Eur. Polym. J..

[35] Lee H.W., Karim M.R., Ji H.M., Choi J.H., Ghim H.D., Park S.M., Oh W., Yeum J.H. (2009). Electrospinning fabrication and characterization of poly(vinyl alcohol)/montmorillonite nanofiber mats. J. Appl. Polym. Sci..

[36] Belozerov A.G., Karasev N.S., Ovchinnikov N.L., Butman M.F. (2014). The effect of preliminary delamination of silicate filler on its exfoliation degree in polyvinyl alcohol/montmorillonite nanocomposites. Nanotechnol. Russ..

[37] Wickham A.M., Islam M.M., Mondal D., Phopase J., Sadhu V., Tamás É., Polisetti N., Richter-Dahlfors A., Liedberg B., Griffith M. (2014). Polycaprolactone-thiophene-conjugated carbon nanotube meshes as scaffolds for cardiac progenitor cells. J. Biomed. Mater. Res. B Appl. Biomater..

[38] Purwar R., Sai Goutham K., Srivastava C.M. (2016). Electrospun Sericin/PVA/Clay nanofibrous mats for antimicrobial air filtration mask. Fibers Polym..

[39] Yuan H., Li B., Liang K., Lou X., Zhang Y. (2014). Regulating drug release from pH- and temperature-responsive electrospun CTS-g-PNIPAAm/poly(ethylene oxide) hydrogel nanofibers. Biomed. Mater..

[40] Vakilian S., Mashayekhan S., Shabani I., Khorashadizadeh M., Fallah A., Soleimani M. (2015). Structural stability and sustained release of protein from a multilayer nanofiber/nanoparticle composite. Int. J. Biol. Macromol..

[41] Raheja A., Agarwal A., Muthuvijayan V., Chandra T.S., Natarajan T.S. (2013). Studies on Encapsulation of Bovine Serum Albumin, Lysozyme and Insulin through Coaxial Electrospinning. J. Biomater. Tissue Eng..

[42] Duque Sánchez L.M., Rodríguez L., López M. (2013). Electrospinning: La era de las nanofibras. Rev. Iberoam. Polímeros.

[43] Pandey J.K., Reddy K.R., Mohanty A.K., Misra M., Pandey J.K., Reddy K.R., Mohanty A.K., Misra M. (2014). Handbook of Polymernanocomposites. Processing, Performance and Application.

[44] Riba J.-R., Esteban B. (2014). A simple laboratory experiment to measure the surface tension of a liquid in contact with air. Eur. J. Phys..

[45] Lee B.-B., Ravindra P., Chan E.-S. (2009). New drop weight analysis for surface tension determination of liquids. Colloids Surf. A Physicochem. Eng. Asp..

[46] Bhardwaj N., Kundu S.C. (2010). Electrospinning: A fascinating fiber fabrication technique. Biotechnol. Adv..

[47] Barzegar F., Bello A., Fabiane M., Khamlich S., Momodu D., Taghizadeh F., Dangbegnon J., Manyala N. (2015). Preparation and characterization of poly(vinyl alcohol)/graphene nanofibers synthesized by electrospinning. J. Phys. Chem. Solids.

[48] Islam M.S., Rahaman M.S., Yeum J.H. (2015). Electrospun novel super-absorbent based on polysaccharide-polyvinyl alcohol-montmorillonite clay nanocomposites. Carbohydr. Polym..

[49] Wang Y., Hsieh Y.-L. (2008). Immobilization of lipase enzyme in polyvinyl alcohol (PVA) nanofibrous membranes. J. Membr. Sci..

[50] Buchko C.J., Chen L.C., Shen Y., Martin D.C. (1999). Processing and microstructural characterization of porous biocompatible protein polymer thin films. Polymer.

[51] Pham Q.P., Sharma U., Mikos A.G. (2006). Electrospinning of Polymeric Nanofibers for Tissue Engineering Applications: A Review. Tissue Eng..

[52] Li Z., Wang C. (2013). One-Dimensional Nanostructures.

[53] Haghi A.K., Akbari M. (2007). Trends in electrospinning of natural nanofibers. Phys. Status Solidi.

[54] Reneker D.H., Chun I. (1996). Nanometre diameter fibres of polymer, produced by electrospinning. Nanotechnology.

[55] Zuo W., Zhu M., Yang W., Yu H., Chen Y., Zhang Y. (2005). Experimental study on relationship between jet instability and formation of beaded fibers during electrospinning. Polym. Eng. Sci..

[56] Zong X., Kim K., Fang D., Ran S., Hsiao B.S., Chu B. (2002). Structure and process relationship of electrospun bioabsorbable nanofiber membranes. Polymer.

[57] Qin Q., Liu Y., Chen S.-C., Zhai F.-Y., Jing X.-K., Wang Y.Z. (2012). Electrospinning fabrication and characterization of poly(vinyl alcohol)/layered double hydroxides composite fibers. J. Appl. Polym. Sci..

[58] Ristolainen N., Heikkilä P., Harlin A., Seppälä J. (2006). Poly(vinyl alcohol) and Polyamide-66 Nanocomposites Prepared by Electrospinning. Macromol. Mater. Eng..

[59] Daga V.K., Helgeson M.E., Wagner N.J. (2006). Electrospinning of neat and laponite-filled aqueous poly(ethylene oxide) solutions. J. Polym. Sci. Part B Polym. Phys..

[60] Zhao C., Qin H., Gong F., Feng M., Zhang S., Yang M. (2005). Mechanical, thermal and flammability properties of polyethylene/clay nanocomposites. Polym. Degrad. Stab..

[61] Lee B.-B., Ravindra P., Chan E.-S. (2008). A critical review: Surface and interfacial tension measurement by the drop weight method. Chem. Eng. Commun..

